# Validation, Invariance, and Reliability of Instruments for the Assessment of Knowledge and Attitudes Toward Cardiopulmonary Resuscitation in Peruvian Children and Adolescents

**DOI:** 10.3390/children12060697

**Published:** 2025-05-29

**Authors:** Ángel López-González, Joseba Rabanales-Sotos, Yrene E. Urbina-Rojas, Zoila E. Leitón-Espinoza, María D. P. Gómez-Luján, Francisco García-Alcaráz, Walter Capa-Luque

**Affiliations:** 1 Nursing College, Universidad de Castilla La Mancha, 02006 Albacete, Spain; angel.lopez@uclm.es (Á.L.-G.); francisco.galcaraz@uclm.es (F.G.-A.); 2 Faculty of Nursing, Universidad Nacional de Tumbes, Tumbes 24001, Peru; yurbina@untumbes.edu.pe; 3 Faculty of Nursing, Universidad Nacional de Trujillo, Trujillo 13011, Peru; zeleiton@unitru.edu.pe (Z.E.L.-E.); mpgomezl@unitru.edu.pe (M.D.P.G.-L.); 4 Faculty of Psychology, Universidad Nacional Federico Villarreal, Lima 15082, Peru; wcapa@unfv.edu.pe

**Keywords:** validation, cardiopulmonary resuscitation, cardiac arrest, knowledge, attitudes, children, adolescents, nursing, reliability, invariance

## Abstract

**Background/Objectives**: In this study, we aimed to analyze the validity, based on the internal structure of the construct, measurement invariance by sex, and reliability of the scores for the “Knowledge in Basic Cardiopulmonary Resuscitation in Peruvian children/adolescents” (KBCPR_P21) and “Attitudes in Basic Cardiopulmonary Resuscitation in Peruvian children/adolescents” (ABCPR_P21) instruments in Spanish. **Methods**: A cross-sectional and instrumental methodological study was conducted between February and August 2021, with the participation of 415 Peruvian elementary school students between 8 and 13 years of age. Participants responded to surveys on knowledge and attitudes toward CPR. For both instruments, exploratory factor analysis (EFA) and confirmatory factor analysis (CFA) was used as the estimation method for categorical data. **Results**: All of the items for both scales have high discriminative capacity (>0.30), and both scales showed high internal consistency (Cronbach’s alpha > 0.87 and McDonald’s omega > 0.90). The validity, based on the internal structure of the construct, implied the existence of a single factor grouping all the items in the two scales (CFI and TLI > 0.95; RMSEA and SRMR < 0.08). Multigroup confirmatory factor analysis also allowed us to satisfactorily verify measurement invariance by sex at the four levels (configural, metric, scalar, and strict) for both scales. **Conclusions**: We can conclude that the values obtained in our evaluation of the scales favor considering them as valid and reliable instruments with which to measure knowledge and attitudes toward basic cardiopulmonary resuscitation in children/adolescents in Peru, given prior learning. The scales could also be used in the evaluation of knowledge and attitudes around basic cardiopulmonary resuscitation in other countries, providing trainers with rapid feedback on the knowledge and attitudes transmitted in training courses, thus allowing better control over the training activities carried out in these courses. Finally, the availability of the scales would allow researchers to empirically test their psychometric properties in other countries.

## 1. Introduction

The world incidence of out-of-hospital cardiac arrest (OHCA) varies greatly between countries, but also between regions within countries; a follow-up study, EuReCa TWO, reported OHCA at 89 per 100,000 inhabitants per year, ranging from 53 to 166, with citizens initiating resuscitation in 58% of cases [1]. The International Liaison Committee on Resuscitation (ILCOR) and its partners urge the teaching of cardiopulmonary resuscitation (CPR) to the entire population in order to provide a rapid and effective response, which will potentially triple the rate of survival from cardiac arrest (CA) if a bystander is able to initiate CPR [2]; therefore, designing strategies through which to teach CPR to the population will increase the chances of survival for an individual in CA [3,4].

The World Health Organization endorsed the Kids Save Lives statement, recognizing that teaching CPR to children and adolescents is an effective way, with a multiplier effect, to increase care for people suffering from CA, which supports its inclusion in the school curriculum [5]. At present, while there is general agreement on the need to teach CPR in schools [6], there is also debate around various aspects of such training, including who should provide it, who to target, how to provide it, from what age, etc. [7].

ILCOR indicates that although simulation is becoming more and more frequently used in the assessment of learning, it is not always easy to objectively assess other transversal competencies that must be acquired; therefore, instruments with which to assess knowledge and to estimate perceived self-efficacy, or each individual’s judgments about his or her abilities, must be made available [8]. It is not enough to “be able to”, but it is also necessary to “feel able to”, to know how to use the knowledge and skills acquired when witnessing a CA [9].

After being trained in CPR, trainees should be assessed on the acquisition of knowledge and skills. They should understand the concepts of CA and CPR, and they should acquire competencies such as the abilities to provide a safe environment, to request help from Emergency Medical Services, to perform chest compressions (proper position, compression site, depth, and frequency), and to open the airway and perform ventilations (mouth-to-mouth) in a patient with/without cervical injury [10,11].

The attitude toward CPR determines the predisposition to react favorably or unfavorably to it, assessing whether, after training, the student feels prepared to perform CPR and, as indicators of having achieved it, whether they feel sufficiently prepared, if the training received has been adequate enough to feel capable, they feel knowledgeable about CPR maneuvers, and they feel able to perform and lead a CPR [12]. The “Knowledge in Basic Cardiopulmonary Resuscitation in Children/Adolescents” (KBCPR_P21) and “Attitudes in Basic Cardiopulmonary Resuscitation in Children/Adolescents” (ABCPR_P21) instruments analyze these aspects [13]; however, these instruments must be validated in terms of content, appearance, criteria, and construct, in addition to satisfying reliable measures [14].

There are different models and instruments that measure the knowledge and emotional responses in both professionals and students of Health Sciences to CPR training [9,14,15], but there are no validated questionnaires that assess whether the child and adolescent population feels able to use their knowledge and skills in the face of a CA after a CPR training process. In this sense, after having performed the content and semantic validations of the instruments for knowledge (KBCPR_P21) and attitudes (ABCPR_P21) regarding basic cardiopulmonary resuscitation [13], the aims of this study were to analyze the validity, based on the internal structure of the construct, measurement invariance by sex, and reliability, of the scores for the Spanish versions of the KBCPR_P21 and ABCPR_P21 instruments in Peruvian children/adolescents.

Their practical applications lie in the availability of useful evaluation instruments for instructors of basic CPR to the population and, likewise, of self-evaluation instruments for the students themselves.

## 2. Materials and Methods

A cross-sectional and instrumental methodological study was conducted as part of the research carried out between February and August 2021, titled “Basic cardiopulmonary resuscitation training project in children and adolescents of a public educational institution”.

This study respected the ethical principles of the Helsinki Convention. The research project was approved by the Research Ethics Committee of the National University of Tumbes, Peru, no. 001-2021/CEI-UNTUMBES. This study had no exclusion criteria.

### 2.1. Participants

A convenience sample of 415 participants was recruited; the inclusion criteria were as follows: students at the ‘8 de octubre’ Primary School in Tumbes (Peru), between 8 and 13 years of age, who accepted voluntary participation in the study, and who had the consent of their parents or guardians. The participants and their legal guardians were informed about the objectives and methodology of the study, as well as their personal contributions to it; in order to participate in the study, participants had to agree and present the signed authorization of their parents or guardians.

### 2.2. Procedures and Instruments

The instruments were applied in a single session in the classrooms of the educational institution by CPR training accredited nurses, after obtaining the informed consent of the children and the consent of their respective parents.

The sociodemographic variables collected from each participant included sex, age, and grade. The scale “Knowledge about Basic Cardiopulmonary Resuscitation in children/adolescents. Peru, 2021 (KBCPR_P21)” (Appendix A) [13] is a self-administered questionnaire that assesses knowledge, created based on the guidelines and protocols of basic CPR 2020 of the American Heart Association [16], and of the European Resuscitation Council 2021 [17]. The questionnaire was composed of eight questions with four answer options, only one of which is correct [18], and all of which were based on previous relevant research [19,20,21,22], with the content validity index for all items being ≥ 0.92, and six of them = 1. The scale is considered to have been passed with correct answers ≥ 80% based on a review of the literature [20,21,22,23,24]. This threshold is based on educational practice for assessing training outcomes but does not impact the psychometric validation of the scale.

The scale “Attitudes about Basic Cardiopulmonary Resuscitation in children/adolescents. Peru, 2021 (ABCPR_P21)” (Appendix B) [13] is a self-administered self-report, consisting of 11 questions with three graduated response options (not capable at all = 1, somewhat capable = 2, fully capable = 3), whose purpose is to identify the attitudes of children/adolescents regarding the care they would offer in the event of CA; these items were selected based on a review of the literature [9,19,21,25], and the content validity of the overall scale was ≥ 0.85 for all items, with nine of them = 1.

### 2.3. Data Analysis

Initially, descriptive analyses of the items were performed to examine item position (mean), response variability (standard deviation), and item distribution (skewness and kurtosis), as well as the discriminative quality of the items (corrected homogeneity index). In accordance with the literature (American Educational Research Association [AERA], the American Psychological Association [APA], and the National Council on Measurement in Education [NCME]), the sample was divided into two random subsamples for the exploratory factor analysis (EFA) and confirmatory factor analysis (CFA).

For both instruments, exploratory factor analysis (EFA) was executed with a sample of 150 children/adolescents (a size that corresponds to the ratio recommended by the literature of 10 respondents for each item of the instrument. The rest of the sample is left for the CFA, which requires a larger sample size); these analyses were performed with the Factor Analysis program, version 12.04.05, for Windows, using the optimal implementation of Parallel Analysis as the estimation method and the Robust Diagonally Weighted Least Squares extraction method, and the factor loadings were estimated from a tetrachoric correlation matrix (KBCPR_P21) and polychoric correlation matrix (ABCPR_P21). In this strategy, the presence of the number of factors or dimensions is evaluated from the explained variance of the first factor, which, to sustain a unifactorial structure, must be greater than 40% [26], in addition to examining the robust goodness-of-fit statistical indices provided by the Factor program, such as the Comparative Fit Index (CFI), Tucker and Lewis Index (TLI), and Root Mean Square Error of Approximation (RMSEA). For the first two indices, values ≥ 0.90 indicate good fit, and values ≥ 0.95 indicate very good fit; for RMSEA, a value ≤ 0.08 indicates adequate fit. In order to rigorously corroborate the validity based on the internal structure of the construct, as well as the measurement invariance of the two instruments, confirmatory factor analysis (CFA) and multigroup CFA were performed on a sample of 265 children/adolescents. R software version 4.3.1 and RStudio 2023.06.2 environment, as well as the lavaan and semTools libraries, were used for these analyses. Given the categorical nature of the items, the estimator used was WLSMV (variance-adjusted weighted least squares) [27]. The recommended CFI, TLI, RMSEA, and RSMR (Standardized Root Mean Square Residual) indices were considered for the assessment of the AFC [28]. Evidence of invariance for each level is satisfied if the difference between the least restrictive model and the immediate most restrictive model for each phase is within the recommended cut-off values (∆CFI < −0.01 and ∆RMSEA < 0.015) [29,30,31]. The evaluation of individual parameters from standardized factor loadings is considered adequate when it is ≥ 0.50 [32].

Finally, reliability was estimated for the instrument scores using the McDonald omega coefficient because its estimation is not biased to the number of response categories in the item or to the number of items, nor does it require compliance with tau equivalence, as occurs with Cronbach’s alpha [33]. An omega coefficient ≥ 0.80 implies that the instrument is of high reliability.

## 3. Results

### 3.1. Psychometric Properties of the Scale for Basic Cardiopulmonary Resuscitation Knowledge in Children/Adolescents (KBCPR_P21)

#### 3.1.1. Item Analysis

Table 1 shows that the data do not follow a normal distribution, with skewness and kurtosis values > ±1.5. It can be identified from the mean that the levels of difficulty range from 0.83 (the easiest item) to 0.19 (the most difficult). The discriminative ability (CHI) of the items in all cases was ≥0.31.

#### 3.1.2. Validity Based on the Internal Structure of the Construct

In the first instance, validity based on the internal structure of the construct for the KBCPR_P21 scale was examined with exploratory factor analysis (KMO = 0.836, Bartlett’s statistic: χ^2^(28) = 1675.1, *p* = 0.000), through which an explained variance for the first factor of 63.05% (eigenvalue = 5.04) and factor loadings (λ) varying between 0.60 and 0.88 (Table 1) were observed; this result of unifactorial structure is supported by the indices for the overall assessment of fit (CFI = 0.968, TLI = 0.955, RMSEA = 0.078).

**Figure 1:**
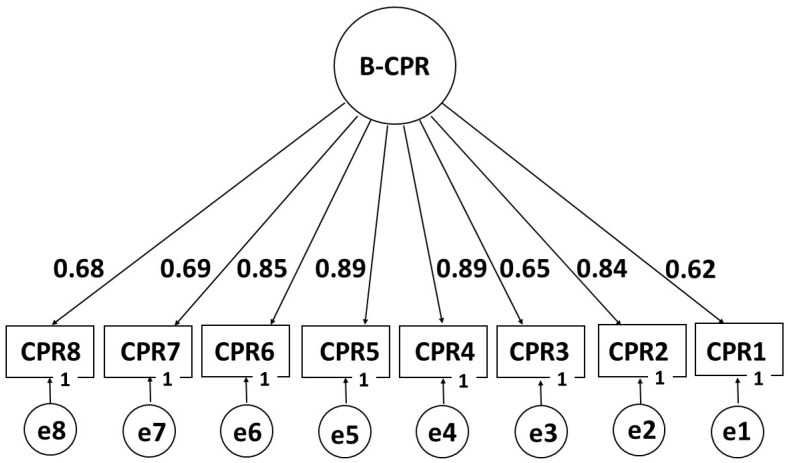
Factor structure of the KBCPR_P21 scale (B-CPR).

Secondly, using the confirmatory factor analysis procedure, the unidimensional structure of the construct was analyzed. As shown in Figure 1, the standardized factor loadings were very good (>0.60). Likewise, the results obtained for the global evaluation of the model were very satisfactory, as follows: χ^2^(20) = 45.725, CFI = 0.981, TLI = 0.974, RMSEA = 0.070, SRMR = 0.074, WRMR = 0.942.

#### 3.1.3. Measurement Invariance by Sex

Table 2 presents the factorial invariance results by sex and age for the KBCPR_P21 scale. The four levels of invariance present adequate fits. As for the nested models (metric, scalar, and strict), the goodness-of-fit indices are within the recommended threshold (∆CFI > −0.01 and ΔRMSEA < 0.015).

#### 3.1.4. Reliability of KBCPR_P21 Scale

For the KBCPR_P21 scale, the internal consistency coefficient estimated with McDonald’s omega (ω = 0.95) denotes high reliability. Likewise, the reliability estimated with the Split-Half technique and corrected with the Spearman–Brown formula is 0.80.

### 3.2. Psychometric Properties of the Scale for Basic Cardiopulmonary Resuscitation Attitudes in Children/Adolescents (ABCPR_P21)

#### 3.2.1. Item Analysis

The mean values (Table 3) indicate that the response options chosen were between 2 and 3. In terms of skewness, the values of the different items varied between −2.04 and −0.29, with those of kurtosis varying between −1.66 and 3.45. The discriminative ability (CHI) in all cases was ≥ 0.38.

#### 3.2.2. Validity Based on the Internal Structure of the Construct

The validity evidence was first evaluated with the exploratory factor analysis strategy to examine the clustering of the items (KMO = 0.795, Bartlett’s statistic: χ^2^(55) = 823.5, *p* = 0.000). A single factor was found to have an explained variance of 44.5% (eigenvalue = 4.89) and the estimated factor loadings (λ) ranged from 0.57 to 0.73 (Table 3); likewise, the overall indices for assessing the fit of the single-factor model were satisfactory (CFI = 0.959, TLI = 0.949, RMSEA = 0.063).

**Figure 2:**
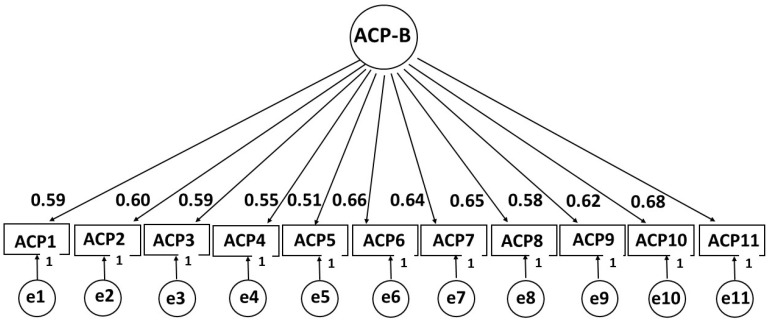
Factor structure of the ABCPR_P21 scale (ACP-B).

A second sample of children/adolescents was used to evaluate a single-factor model with the confirmatory factor analysis strategy. As shown in Figure 2, the standardized factor loadings varied between good (0.51) and very good (0.68). All of the overall fit indices of the model were very adequate, with χ^2^(43) = 85.697, CFI = 0.975, TLI = 0.968, RMSEA = 0.063, SRMR = 0.078.

Therefore, the results found support construct validity for the ABCPR_P21 scale score.

#### 3.2.3. Measurement Invariance by Sex and Age

Once the factor structure of the ABCPR_P21 scale was established, the measurement invariance according to sex and age was analyzed. Table 4 shows that the configural invariance presents an adequate fit (CFI > 0.95 and RMSEA < 0.08). As for the nested models (metric, scalar, and strict), the recommended values of ∆CFI (>−0.01) and ΔRMSEA (<0.015) denote compliance with invariance by both sex and age.

#### 3.2.4. Reliability of ABCPR_P21 Scale

For the ABCPR_P21 scale, internal consistency reliability was estimated for categorically graded items with McDonald’s omega (ω = 0.90), the coefficient of which denoted high reliability. Likewise, the reliability estimated with the Split-Half technique and corrected with the Spearman–Brown formula is 0.79.

## 4. Discussion

The aim of this study was to examine the evidence of validity, based on the internal structure of the construct, measurement invariance by sex and age, and reliability of the scores for the KBCPR_P21 and ABCPR_P21 instruments. This work arose from the need for validated instruments to assess knowledge and attitudes around basic CPR in children/adolescents attending public schools in Peru, as there were no comparable instruments in the literature according to the country’s training curriculum with regard to the ages of the students [9,19,20,21,22,31,34], with the exception of the ones published by Gutiérrez-Sánchez B et al. and Borovnik Lesjak et al., in which they evaluated the knowledge of students of similar ages [35,36,37].

Our final analysis shows that, in the KBCPR_P21 questionnaire, items have high discriminative capacity (IHC > 0.30) to assess knowledge, identifying CPR1 (What is a heart arrest?) as the easiest item (0.83) and CPR6 (If, after some time performing compressions in the center of the person’s chest, I verify that he/she is still unconscious and not breathing, what should I do?) as the most difficult (0.19), with most items being of medium difficulty (≥0.40), a result comparable to that reported by Banfai et al. in this age group [23]. The McDonald omega score (ω = 0.95) is considered, according to available research, to be a value of high reliability [33].

The validity, based on the internal structure of the construct for the KBCPR_P21 scale, implied the existence of a single factor grouping all of the items; this result of unifactorial structure is supported by the indices for the overall assessment of fit (CFI = 0.968, TLI = 0.955, RMSEA = 0.078).

The overall evaluation of the model presented very satisfactory fit indices and high individual parameters expressed in the factor loadings, results which support satisfactory evidence of validity based on the internal structure of the construct for the KBCPR_P21 scale.

The results of factorial invariance by sex and age for the KBCPR_P21 scale were not affected by the progressively imposed restrictions since they did not deteriorate the fit of the models examined with the four levels of invariance. The configural model (the base model without equality restrictions for parameter estimation) presents adequate fit, which evidences the existence of the same unifactorial model for males and females, without age differentiation. For the nested models, with restrictions in each phase, the suggested fit indices (∆CFI > −0.01 and ΔRMSEA < 0. 015) are within the appropriate threshold [9,16,17], meeting the metric invariance (with factor loading restriction), which means that the latent construct has the same meaning without distinction of sex and age; likewise, the scalar variance (with restrictions on factor loadings and intercepts) evidences that the indicator score and the KBCPR_P21 scale latent score are equivalent for both boys and girls, as well as for children from 8 to 10 years old and 11 to 13 years old. Finally, the strict invariance (with restrictions on factor loadings, intercepts, and residuals) evidences that the items are measured with the same precision in each group by gender and age.

Our research agrees with that of Pîvac et al. and Bohn et al. [24,38], and we propose knowledge of how to act when faced with an unconscious person (CPR2), what to communicate when calling the Emergency Medical Services (EMS) (CPR3), how to act when faced with a person in CA (CPR4, CPR5), what a CA is, why chest compressions should be given to a person in CA, and what to do when the EMS arrive (CPR1, CPR6, CPR7, and CPR8) as items to be measured. The simplicity of the questions asked in the investigated scale finds justification in what was contributed by Meissner et al., Naqvi et al., and Lukas et al. [21,39,40], in that the level of CPR knowledge was higher in children with a mean age of 14.5 years when compared to that of younger children. After the implemented CPR training, the level of knowledge increased in all age groups.

In the ABCPR_P21 questionnaire, a self-report of typical performance measuring attitudes, participants mostly indicated response options between 2 (somewhat capable) and 3 (very capable) after receiving CPR training. In terms of skewness and kurtosis, there were items with values > ±1.5, indicating that the data do not follow a normal distribution. The IHC values show that all items have high discriminative capacity (CHI > 0.30); the McDonald’s omega score (ω = 0.90) is considered a value of high reliability [33].

The validity, based on the internal structure of the construct for the ABCPR_P21 scale, implied the existence of a single factor grouping all the items. The global indices for assessing the fit of a one-factor model supporting the unidimensionality of the construct include CFI = 0.959, TLI = 0.949, and RMSEA = 0.063.

The overall fit indices found to support the existence of a unifactorial structure were very satisfactory; likewise, the standardized factor loadings were considered high (λ > 0.50). Consequently, it can be stated that the results support the existence of validity for the ABCPR_P21 scale score based on its internal structure.

The configural invariance presents adequate fit because CFI and RMSEA satisfy the recommended parameters. As for the nested models, the ∆CFI (>−0.01) and ΔRMSEA (<0.015) satisfy the recommended critical values for the compliance of metric, scalar, and strict variance [23,24,25], results evidencing that, for both males and females, as well as for children (8 to 10 years old) and adolescents (11 to 13 years old), the factor structure is the same. Similarly, the constructs measured have the same meanings and are measured with the same precision.

The ABCPR_P21 questionnaire asks children/adolescents about their willingness, attitudes, and intentions to help a person in CA after receiving CPR training. The items included ask about their self-esteem and moral responsibility toward themselves and the people around them [39,40]. The application of the questionnaire will inform the influence of CPR training on the intention to help others and assess what was achieved by implementing early and age-sequenced CPR training in terms of the improvement of schoolchildren’s attitudes and intentions to help others, their willingness to do so, and their ability to express empathy [41,42,43].

Some strengths of the research presented herein are the robustness of the validity based on the internal structure of the construct, the relevant reliability for categorical items, and the measurement invariance that allows for the evaluation and derivation of inferences from the scores in the two questionnaires in a similar way for males and females.

Future research will be needed to test whether unsatisfactory attitudinal responses and failure to achieve adequate knowledge scores can be considered an opportunity to increase the interest of schoolchildren in participating in theoretical–practical CRP recycling; it will also be in the revision of the training curricula and in the adequate selection of trained teachers to manage the teaching process so that it supports the needs of the group of students in a way that connects as much as possible with their interests, sense of willingness, and current level of knowledge.

Currently, the educational curricula in various countries provide theoretical and practical CPR content in the initial stages of education. Nursing, in its responsibility to educate the population and disseminate health education actions, is key in this teaching process. The availability of scales that assess the attitudes and knowledge of those who have attended the training courses will be essential to understanding the quality of the teaching provided, what the students have learned, and whether they are prepared to intervene in an OHCA or other emergency situation from an early age and according to their abilities.

Several limitations should be taken into account in this study. Firstly, the scarcity of instruments found in the scientific literature that could be used to assess knowledge and attitudes in the child/adolescent population obliged a group of experts to develop the questionnaires and validate them semantically. Another limitation may lie in the fact that assessing only theoretical knowledge is not the only option, so we suggest further research focused on the acquisition of practical knowledge and its influence on the attitudes of schoolchildren towards CA. Finally, the present study was developed in one Public Primary School of Tumbes, Peru, so for the administration of the scales in other socio-economically different settings in Peru or in other Spanish-speaking countries, a cross-cultural validation is suggested [44].

## 5. Conclusions

The values obtained in our validations of the KBCPR_P21 and ABCPR_P21 instruments are in favor of considering them as valid and reliable instruments with which to measure knowledge and attitudes toward basic CPR in children/adolescents in Peru after prior learning. The KBCPR_P21 and ABCPR_P21 scales could also be used in the evaluation of knowledge and attitudes around basic CPR in other countries, in order to provide CPR trainers with rapid feedback on the knowledge and attitudes transmitted in basic CPR courses, thus allowing for better control over the training activities carried out. Finally, the availability of the KBCPR_P21 and ABCPR_P21 scales will allow researchers to empirically test their psychometric properties in other countries.

In practical application, this research provides useful instruments for the evaluation of learning and self-evaluation in basic CPR that can be used in continuing education in CPR for schoolchildren as part of the compulsory school curriculum.

## Figures and Tables

**Table 1:** Item analysis for the KBCPR_P21 scale (sample = 415).

	M	SD	Skewness	Kurtosis	CHI	λ
CPR1	0.83	0.37	−1.81	1.27	0.31	0.67
CPR 2	0.33	0.47	0.72	−1.49	0.61	0.88
CPR 3	0.63	0.48	−0.56	−1.70	0.45	0.60
CPR 4	0.39	0.49	0.44	−1.82	0.64	0.84
CPR 5	0.40	0.49	0.39	−1.86	0.66	0.85
CPR 6	0.19	0.39	1.60	0.57	0.51	0.84
CPR 7	0.63	0.48	−0.54	−1.72	0.47	0.63
CPR 8	0.50	0.50	−0.01	−2.02	0.48	0.76

Note: M: mean; SD: standard deviation; CHI: corrected homogeneity index; λ: factor loadings.

**Table 2:** Factorial invariance of the KBCPR_P21 scale (B-CPR) by sex and age (sample = 265).

Models	χ^2^	df	*p*	CFI	ΔCFI	RMSEA	ΔRMSEA
**Sex**							
Configural	53.921	40	0.07	0.981		0.051	
Metric	66.659	47	0.03	0.973	−0.008	0.056	0.005
Scalar	75.885	54	0.02	0.969	−0.004	0.056	0.000
Strict	86.262	62	0.02	0.966	−0.003	0.055	−0.001
**Age**							
Configural	70.093	40	0.002	0.958		0.057	
Metric	58.106	47	0.128	0.985	0.027	0.042	−0.015
Scalar	69.532	54	0.076	0.979	−0.006	0.047	0.005
Strict	73.364	62	0.153	0.984	0.005	0.037	−0.010

Note: χ^2^= Chi squared, df = degrees of freedom, *p* = probability of significance; CFI: Comparative Fit Index; RMSEA: Root Mean Square Error of Approximation.

**Table 3:** Item analysis for the ABCPR_P21 scale (ACP-B) (sample = 415).

Items	M	SD	Skewness	Kurtosis	CHI	λ
ACP1	2.68	0.52	−1.32	0.75	0.43	0.70
ACP2	2.56	0.57	−0.86	−0.27	0.45	0.59
ACP3	2.59	0.52	−0.71	−0.78	0.43	0.62
ACP4	2.70	0.49	−1.30	0.58	0.39	0.62
ACP5	2.56	0.51	−0.34	−1.66	0.38	0.60
ACP6	2.40	0.57	−0.29	−0.76	0.49	0.59
ACP7	2.62	0.56	−1.12	0.28	0.47	0.58
ACP8	2.79	0.46	−2.04	3.45	0.46	0.57
ACP9	2.70	0.48	−1.10	−0.23	0.44	0.73
ACP10	2.74	0.46	−1.33	0.40	0.43	0.66
ACP11	2.67	0.51	−1.18	0.32	0.48	0.72

Note: M: mean; SD: standard deviation; CHI: corrected homogeneity index; λ: factor loadings.

**Table 4:** Factorial invariance of the ABCPR_P21 scale (PCA-B) by sex and age (sample = 265).

	χ^2^	df	*p*	CFI	ΔCFI	RMSEA	ΔRMSEA
**Sex**							
Configural	139.51	88	0.000	0.959		0.069	
Metric	138.958	98	0.004	0.959	0.000	0.058	−0.011
Scalar	147.001	108	0.008	0.953	−0.006	0.054	−0.004
Strict	156.003	119	0.013	0.955	0.002	0.050	−0.004
**Age**							
Configural	125.065	88	0.004	0.922		0.061	
Metric	121.271	98	0.042	0.949	0.027	0.046	−0.015
Scalar	126.887	108	0.082	0.958	0.009	0.040	−0.006
Strict	144.182	119	0.045	0.966	0.008	0.043	0.003

Note: χ^2^= Chi squared, df = degrees of freedom, *p* = probability of significance; CFI: Comparative Fit Index; RMSEA: Root Mean Square Error of Approximation.

## Data Availability

The dataset is available on request from the authors. The data is not publicly available for legal and ethical reasons.

## Abbreviations

The following abbreviations are used in this manuscript:

ABCPR_P21	Attitudes in Basic Cardiopulmonary Resuscitation in Children/Adolescents
CA	Cardiac arrest
CFA	Confirmatory factor analysis
CFI	Comparative Fit Index
CPR	Cardiopulmonary resuscitation
EFA	Exploratory factor analysis
EMS	Emergency Medical Services
ILCOR	International Liaison Committee on Resuscitation
KBCPR_P21	Knowledge in Basic Cardiopulmonary Resuscitation in Children/Adolescents
RMSEA	Root Mean Square Error of Approximation
WLSMV	Variance-adjusted weighted least squares
TLI	Tucker and Lewis Index

## Appendix A

Knowledge about Basic Cardiopulmonary Resuscitation in children/adolescents. Peru, 2021 (KBCPR_P21)

**1- ¿Qué es un paro cardiaco?** a) El corazón deja de latir y la persona no respira. b) El corazón lentamente deja de latir. c) Falta de la respiración. d) Pérdida brusca de la conciencia. **1- What is heart arrest?** a) The heart stops beating and the person is not breathing b) The heart slowly stops beating. c) Lack of breathing. d) Abrupt loss of consciousness	**5- Si después de realizar las compresiones en el centro del pecho de las personas, CONFIRMO QUE RESPIRAN, ¿qué debo hacer?** a) Lo siento y le doy agua. b) Lo pongo de costado. c) Le cuento lo que le sucedió. d) Lo llevo a un lugar más fresco. **5- If after doing the compressions in the centre of the person’s chest I CONFIRM THEY ARE BREATHING** a) I sit him down and give him water. b) I put him on its side. c) I tell him what happened. d) I take him to a cooler place.
**2- ¿De los siguientes, ¿cuál es el primer paso a ser realizado frente a una persona con pérdida de la conciencia?** a) Pedir ayuda al servicio de salud llamando al 116 *. b) Extender el cuello hacia atrás para que el aire entre. c) Mover a la persona inconsciente para que despierte. d) Estar al lado de la persona hasta que llegue la ambulancia. **2- Of the following, what is the first step to be taken in front of a person with loss of consciousness?** a) Request help from the health service by calling 116 *. b) Extend the neck backwards so that the air enters. c) Move the unconscious person to wake up. d) Be by the person’s side until the ambulance arrives.	**6- Si después de un tiempo realizando compresiones en el centro del pecho, la persona sigue inconsciente y sin respirar ¿Qué debo hacer?** a) Continuaré con las compresiones hasta llegar la ayuda. b) Pido que una persona ayude y me pongo a filmar. c) Pido que otra persona haga las compresiones mientras descanso. d) Continuo las compresiones, aunque me encuentre muy cansado. **6- If after some time doing chest compressions on the centre of the chest, the person is still unconscious and not breathing, what should I do?** a) I will continue with compressions until help arrives. b) I ask someone to help, and I start filming. c) I ask someone else to do the compressions while I rest. d) I continue compressions, even if I feel very tired.
**3- ¿Qué debo decir cuando llamo por teléfono al número de Emergencia, por ejemplo, a los bomberos 116?** a) Informo que la persona no responde, no respira y donde me encuentro. b) Grito informando mis datos pidiendo ayuda y cuelgo inmediatamente. c) Pido que otra persona pase las informaciones sobre lo que está sucediendo con la persona. d) Grito y pido ayuda que otra persona pase las informaciones al personal de salud. **3- What should I say when I call the emergency number, for example, the fire department 116?** a) I inform that the person does not respond, is not breathing and where I am. b) I shout reporting my data asking for help and hang up immediately. c) I ask another person to pass on the information about what is happening with the person. d) I scream and ask someone else to pass the information to the health personnel.	**7- ¿Por qué se debe hacer compresiones en el centro del pecho en la persona en paro cardiaco?** a) Porque con cada compresión estimula al corazón y llega la sangre a todo el cuerpo. b) Para que se despierte, camine y vaya a casa. c) Porque el aire entra mejor a los pulmones. d) Porque la persona puede estar atragantada y de ese modo el cuerpo extraño puede salir. **7- Why should compressions be given to the centre of the chest in the person in cardiac arrest?** a) Because with each compression it stimulates the heart and blood reaches the entire body. b) For him to wake up, walk and go home. c) Because the air enters the lungs better. d) Because the person may be choking and thus the foreign body may come out.
**4- ¿Mientras llegue la ambulancia qué ritmo de compresiones debo realizar en el centro del pecho de la persona?** a) 30 compresiones por minuto. b) 15 compresiones por minuto. c) 100–120 compresiones por minuto. d) Las necesarias hasta llegar la ambulancia. **4- While the ambulance arrives, what rhythm compressions should I perform in the centre of the person’s chest?** a) 30 compressions per minute. b) 15 compressions per minute. c) 100–120 compressions per minute. d) Those necessary until the ambulance arrives	**8- ¿Qué debemos hacer cuando llegue la ambulancia?** a) Me retiro y me voy a mi casa. b) Comienzo a filmar con mi celular. c) Explicarle lo qué se ha hecho hasta su llegada. d) Acompañar a la persona al hospital. **8- What should we do when the ambulance arrives?** a) I retire and go home. b) I start filming with my cell phone. c) Explain to him what has been done until his arrival. d) Accompany the person to the hospital.
* 116 is the telephone number of the Fire Brigade in Peru.	

The English translation is not validated, it is only an approximation to facilitate the understanding of the basis of the research.

ANSWER TEMPLATE

1: A; 2: A; 3: A; 4: D; 5: B; 6: A; 7: A; 8: C

## Appendix B

Attitudes about Basic Cardiopulmonary Resuscitation in children/adolescents. Peru, 2021 (ABCPR_P21)

**Items**	**Me siento** ***I feel***		
	**Muy capaz** ***Very able*** **Fully capable**	**Algo capaz** ***Somewhat* capable**	**Muy poco capaz** **Not capable al all**
1. Me siento capaz y estoy preparado para reaccionar ante una de emergencia en la que la vida peligra. *1. I feel able and prepared to react to an emergency in which life is in danger.*			
2. Me siento capaz de mantener la calma cuando me encuentre ante una persona que se ha desmayado y NO respira. *2. I am able to remain calm when faced with a person who has fainted and is NOT breathing.*			
3. Me siento capaz de tomar decisiones cuando me encuentre ante una persona que se ha desmayado y NO respira. *3. I feel capable of making decisions when I am faced with a person who has fainted and is NOT breathing.*			
4. Me siento capaz de llamar rápidamente a algún número de emergencia, por ejemplo, a los bomberos (116), cuando encuentro a una persona que se ha desmayado y NO respira. *4. I feel able to quickly call an emergency number, for example, the fire department (116), when I find a person who has fainted and is NOT breathing.*			
5. Me siento capaz de informar de los detalles al telefonista de emergencia de forma tranquila *5. I feel able to calmly report details to the emergency operator.*			
6. Me siento capaz de abrir la vía aérea en una persona inconsciente. *6. I feel able to open the airway in an unconscious person.*			
7. Me siento capaz de realizar las compresiones en el centro del pecho a una persona desconocida en paro cardiaco. *7. I feel able to perform center chest compressions on an unknown person in cardiac arrest.*			
8. Me siento capaz de realizar las compresiones en el centro del pecho a un familiar cercano en paro cardiaco. *8. I feel able to perform center chest compressions on a close family member in cardiac arrest.*			
9. Me siento capaz de informar a los profesionales de salud del procedimiento realizado en la persona en paro cardiaco. *9. I feel capable of informing the health professionals of the procedure performed on the person in cardiac arrest.*			
10. Me siento capaz de APRENDER la Resucitación Cardiopulmonar (RCP) porque con ella puedo salvar vidas. *10. I feel able to LEARN Cardiopulmonary Resuscitation (CPR) because with it I can save lives.*			
11. Me siento capaz de REALIZAR la Resucitación Cardiopulmonar (RCP) porque con ella puedo salvar vidas. *11. I feel able to PERFORM Cardiopulmonary Resuscitation (CPR) because with it I can save lives.*			
The English translation is not validated, it is only an approximation to facilitate the understanding of the basis of the research.			
